# *Neisseria meningitidis* Induced Fatal Waterhouse–Friderichsen Syndrome in a Patient Presenting With Disseminated Intravascular Coagulation and Multiple Organ Failure

**DOI:** 10.3390/brainsci10030171

**Published:** 2020-03-17

**Authors:** Meng-Yu Wu, Chien-Sheng Chen, Chih-Yi Tsay, Giou-Teng Yiang, Jian-Yu Ke, Po-Chen Lin

**Affiliations:** 1 Department of Emergency Medicine, Taipei Tzu Chi Hospital, Buddhist Tzu Chi Medical Foundation, New Taipei City 231, Taiwan; skyshangrila@gmail.com (M.-Y.W.); holeyeye@yahoo.com.tw (C.-S.C.); belle99311101@gmail.com (C.-Y.T.); gtyiang@gmail.com (G.-T.Y.); 2 Department of Emergency Medicine, School of Medicine, Tzu Chi University, Hualien 970, Taiwan

**Keywords:** *Neisseria meningitidis*, meningococcemia, Waterhouse–Friderichsen syndrome, disseminated intravascular coagulation, multiple organ failure

## Abstract

*Neisseria meningitidis*-induced acute systemic meningococcal disease is an emergency and a fatal condition that has a high mortality rate. In patients with a fulminant infection, a maculopapular petechial eruption, purpura fulminans, or an ecchymotic lesion are worrisome signs reflecting disseminated intravascular coagulation (DIC) and hint at Waterhouse–Friderichsen syndrome (WFS). Here, we describe a rare case of a patient with a fulminant *Neisseria meningitidis-*induced acute systemic meningococcal disease presenting with high-grade fever without meningitis symptoms. Fatal septicemia with DIC and multiple organ failure was noted. WFS was chiefly suspected. We highlight the clinical features and pathogenesis of *Neisseria meningitidis*-induced meningococcemia and WFS. We propose that they should be kept in mind, especially in patients presenting with a petechial eruption and purpura fulminans.

## 1. Introduction

*Neisseria meningitidis*-induced acute systemic meningococcal disease is an emergency and a fatal condition that has a high mortality rate [1]. Clinical manifestations of meningococcal meningitis and/or accompanying meningococcemia are commonly seen in patients with *Neisseria meningitidis* infection. However, absence of clinical evidence of meningitis in patients presenting with meningococcemia may lead to missed diagnosis and lack of timely treatment. In patients with a fulminant infection, a maculopapular petechial eruption, purpura fulminans, or an ecchymotic lesion may be seen. Severe sepsis may also induce disseminated intravascular coagulation (DIC) and Waterhouse–Friderichsen syndrome (WFS) described as acute hemorrhagic necrosis of the adrenal glands. These rare clinical features predict high morbidity and mortality [2]. Even with timely intervention, patients with *Neisseria meningitidis*-induced WFS usually die within 24 h. Therefore, early diagnosis is very important for physicians for timely intervention in *Neisseria meningitidis*-induced acute systemic meningococcal disease and administration of antibiotics. Here, we describe a rare case of a fulminant *Neisseria meningitidis*-induced acute systemic meningococcal disease presenting with meningococcemia without meningitis. The patient had severe sepsis-induced DIC and multiple organ failure. WFS was chiefly suspected. The clinical features and pathogenesis of *Neisseria meningitidis* meningococcemia and WFS are discussed.

## 2. Case Presentation

**Figure 1:**
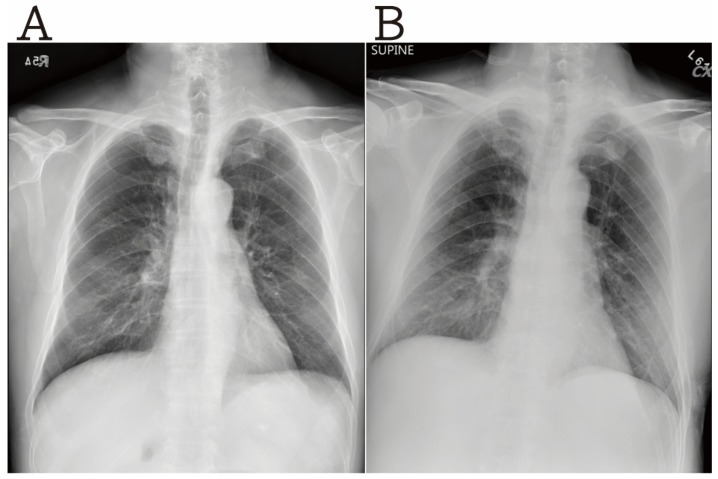
The chest X-ray revealed mild increased bilateral lung markings without a significant pneumonia patch (**A**) initially (**B**) after one day.

A 67-year-old man presented with high-grade fever (39.0 °C) for a day. He had a past medical history of hypertension and cardiac arrhythmia, but denied having a history of diabetes mellitus, immunosuppression, or malignancy. Initial symptoms included high-grade fever and general muscle soreness. The patient did not have a headache, dyspnea, nausea, vomiting, abdominal pain, diarrhea, dysuria, chest pain, or a skin rash. Additional symptoms, such as hemorrhagic tendency/abnormality or arthritis, were not found. He had a history of traveling to Kinmen, Taiwan. However, he did not come in contact with other people with similar symptoms. On physical examination, his temperature was 39 °C, blood pressure was 142/85 mm Hg, heart rate was 101 beats/min, and the Glasgow Coma Score (GCS) was E_4_V_5_M_6_. The breath sound was clear, and abdomen was soft without local tenderness. There were no abnormal skin lesions or neurological signs. The initial laboratory evaluation revealed no significant infection pattern, and the influenza test was negative (Table 1). The chest X-ray showed no significant pneumonia patch (Figure 1A). During observation at the ER, there were no progressive dyspnea or red flag signs. He received supportive care and was discharged. However, one day later, he revisited our hospital due to persistent high-grade fever and new onset of subconjunctival hemorrhage with petechial skin rash over the trunk and extremities. Other symptoms included epigastric pain and vomiting. Shock was suspected on the vital signs assessment: temperature was 38.8 °C, blood pressure was 107/66 mm Hg, and heart rate was 98 beats/min. The secondary laboratory evaluation revealed severe sepsis, acute kidney injury, and metabolic acidosis. Low platelet count, prolonged prothrombin time (PT), and activated partial thromboplastin time (APTT) reflected severe DIC. We suspected severe complications of the influenza virus infection based on the clinical symptoms. However, the secondary influenza test was negative, as well as the dengue test. Abdominal computed tomography (CT) scanning was arranged to rule out an intraabdominal infection. Even with early administration of ceftriaxone and levofloxacin, progressive cyanosis at bilateral fingers and lips was found along with dyspnea. The patient experienced a sudden cardiac arrest. Despite cardiopulmonary resuscitation (CPR), he expired. Subsequently, the blood culture report showed *Neisseria meningitidis.* This result was confirmed by the Centers for Disease Control and Prevention (CDC), who detected *Neisseria meningitidis* serogroup B.

## 3. Discussion

*Neisseria meningitidis* is a gram-negative human-specific pathogen classified into thirteen serotypes [3]. Serotypes A, B, C, Y, and W135 are the major disease-causing serogroups. In the United States, serotype B causes about one-third of all invasive diseases [4]. The clinical manifestations of *Neisseria meningitidis* could range from transient fever with non-specific symptoms to invasive and fulminant diseases, such as meningitis and meningococcemia. The clinical presentation of meningitis includes the classic symptoms: headache, neck stiffness, and fever. Other non-specific symptoms are nausea, vomiting, myalgia, and poor appetite. In the present case, only flu-like symptoms were found. He denied any significant symptoms of urinary tract infection, acute intraabdominal infection, and meningitis. In a study by Rodrigo Siqueira Batista et al. [5], symptoms such as sore throat, coryza, cough, and otalgia were found after the incubation period. The classic symptoms occurred only in 44% of patients and were also more common in *Streptococcus pneumoniae* infection [6]. These atypical symptoms could be misdiagnosed as a viral infection, especially influenza. Skin rash, such as a petechial, maculopapular eruption, an ecchymotic lesion, and purpura fulminans are rare hallmarks of an invasive meningococcal disease. In the current patient, skin rash developed within 24 h, and the condition was compatible with the previous data which reflected a worse prognosis. Other clinical features of worse prognosis included extreme age, primary meningococcal pneumonia, shock status, loss of consciousness, and seizures [7]. In addition, disease-promoting comorbidities such as immunosuppression could lead to missing the important symptoms or induce sepsis progression. In a report by Nicolas Paleiron et al. [8], a patient with a fulminant *Neisseria meningitidis* B infection was found with multiple myeloma, illustrating the importance of immunosuppression in *Neisseria meningitidis* infection. However, this patient did not have significant immunosuppressive diseases.

Purpura fulminans was characterized as vascular thrombosis with hemorrhagic necrosis of the skin with formation of bullae and vesicles. The necrosis can follow with extension into the subcutaneous tissue, muscles and bones. These skin rashes were clinical indicators of the potential bleeding complications secondary to thrombocytopenia and disseminated intravascular coagulation (DIC). Progressive meningococcal sepsis also induced WFS, which is defined as an acute adrenal failure related to acute hemorrhagic necrosis of the adrenal glands. The typical symptoms of WFS include fever, maculopapular skin rash, petechiae, ecchymosis, cyanosis of the extremities, and shock resulting from severe sepsis via coagulopathy [9]. It could also be caused by other bacteria, such as *Proteus Mirabilis, Pseudomonas aeruginosa*, *Streptococcus pneumoniae*, *Mycobacterium tuberculosis*, and *Haemophilus influenzae* [10,11]. WFS is a catastrophic syndrome, and it is difficult to be diagnosed in the clinical course due to hypotension and quick progression to shock. Adrenal hemorrhage is usually confirmed during the autopsy [12]. In the present case, an abdominal CT scan was arranged for high suspicion of WFS due to the typical clinical features. However, the progressive shock status could not be corrected even after aggressive resuscitation.

**Figure 2:**
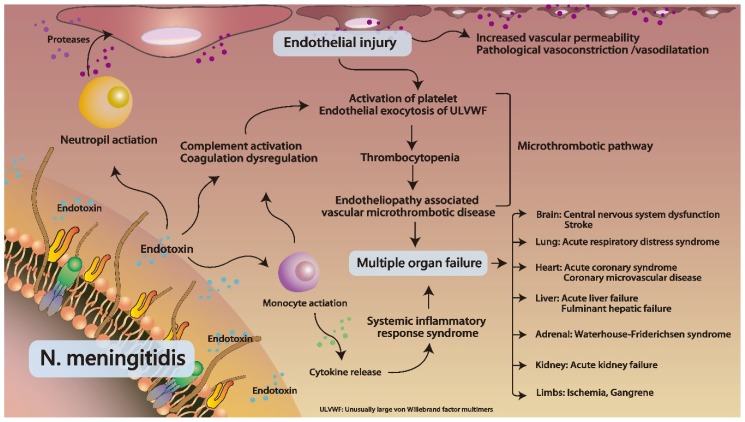
A schematic diagram illustrating the detailed pathophysiological mechanism between septicemia and disseminated intravascular coagulation in *Neisseria meningitidis* infection. Adapted from Chang [14,15] and Pathan et al. [13].

The pathophysiology of a meningococcemia-induced WFS remained unclear. In a study by Pathan et al. [13], *Neisseria meningitidis* may adhere to non-ciliated epithelial cells and avoid host immune systems by releasing the IgA protease, presenting with asymptomatic nasopharyngeal colonization. After invasion into the bloodstream, the endotoxin of *Neisseria meningitidis* may trigger immune cell activation, including the monocytes and neutrophils. Neutrophils can release the proteases causing endothelial injury. Monocytes may promote cytokines to induce the systemic inflammatory response syndrome. In addition, the endotoxin may also activate the complement system and dysregulate the coagulation factors. According to a summary by Chang et al. [14], these are effects of dysregulation of the immune system causing endotheliopathy, leading to the activation of the inflammatory pathway and the microthrombotic pathway. This causes a cytokine storm, disseminated intravascular microthrombosis, and endotheliopathy-associated vascular microthrombotic disease. Finally, the patients may present with multiple organ failure and thrombotic thrombocytopenic purpura-like syndrome, such as WFS and purpura fulminans (Figure 2).

The prognosis of *Neisseria meningitidis* infection depends on its severity and treatment. The mortality of WFS is approximately 20%, rising to more than 50% when there is shock. Early diagnosis and timely treatment of meningococcal sepsis is important to reduce the mortality. In pediatric or adult patients with an invasive meningococcal disease, the third generation of cephalosporin, ceftriaxone, was suggested to be the antibiotic of choice for *Neisseria meningitidis* infection. If patient was intolerant or allergic to beta-lactam antibiotics, chloramphenicol was an acceptable alternative medication [16]. In a severe invasive meningococcal disease, timely correction of the shock status is a critical step including rapid stabilization of the airways and breathing, intravenous access, and adequate fluid resuscitation [17]. Corticosteroids were considered for WFS due to the adrenal insufficiency status. Hydrocortisone 200 mg daily has been recommended. In the current case, the flu-like symptoms without significant infection signs may have contributed to different diagnosis. When the skin rash erupted and progressive shock status occurred, *Neisseria meningitidis*-induced WFS was highly suspected. Despite our attempt to resuscitate, the fulminant sepsis could not be corrected even with administration of ceftriaxone. We present a rare case of *Neisseria meningitidis*-induced WFS to highlight an uncommon but important differential diagnosis that should be kept in mind in patients with a petechial, maculopapular eruption, or an ecchymotic lesion. Early management can prevent the poor clinical prognosis.

## Figures and Tables

**Table 1:** The laboratory evaluation of this patient.

Variables	Normal Range	Patient Data		Variables	Normal Range	Patient Data	
		Day 1	Day 2			Day 1	Day 2
White cell count	3.9–10.6 (× 103/µL)	8.69	2.19	Blood urine nitrogen	7–25 mg/dL	17	26
Hemoglobin	13.5–17.5 g/dL	15.1	15.5	Creatinine	0.7–1.3 mg/dL	1.1	2.7
Platelet count	150–400 (× 103/µL)	152	35	Sodium	136–145 mmol/L	133	134
Band	0–3%	1.0	6.0	Potassium	3.5–5.1 mmol/L	3.6	4.0
Monocytes	2–10%	1.0	4.0	Glucose	70–100 mg/dL	90	99
Neutrophils	40–45%	95.0	62.0	ALT	7–52 U/L	---	28
Lymphocytes	20–45%	3.0	24.0	AST	13–39 U/L	---	44
Eosinophils	1–6%	0.0	1.0	Total bilirubin	0.3–1.0 mg/dL	---	2.08
N. RBC	0–0% (/100 WBC)	0.0	7.0	C-reactive protein	< 1.0 mg/dL	0.23	10.33
Metamyelocytes	0–0%	0.0	2.0	Lactic acid	0.5–2.2 mg/dL	---	11.3
PT	8–12 s	---	28.2	Hs-troponin I	< 17.5 pg/mL	---	64.8
APTT	23.9–35.5 s	---	166.2	Influenza test	Negative	Negative	Negative
INR		---	2.77	Dengue test*	Negative	Negative	Negative
Venous gas pH	7.31–7.41	---	7.239				
Venous gas pCO_2_	41–51 mm Hg	---	41.7				
Venous gas pO_2_	80–100 mm Hg	---	33.6				
Venous gas HCO_3_^-^	22–26 mmol/L	---	17.4				

N. RBC: Nucleated red blood cells; PT: Prothrombin time; APTT: Activated partial thromboplastin time; ALT: Alanine aminotransferase; AST: Aspartate aminotransferase; Dengue test*: included NS1 antibodies, IgM, and IgG; INR: international normalized ratio.
